# Gallic acid enhances anti-lymphoma function of anti-CD19 CAR-T cells *in vitro* and *in vivo*

**DOI:** 10.1186/s43556-023-00122-6

**Published:** 2023-03-05

**Authors:** Zhiqiang Luo, Jiaru Shi, Qiyao Jiang, Guohua Yu, Xiaorui Li, Zhuoying Yu, Jianxun Wang, Yuanyuan Shi

**Affiliations:** 1 School of Life Sciences, Beijing University of Chinese Medicine, Beijing, 102488 China; 2 State Key Laboratory of Dao-di Herbs, National Resource Center for Chinese Materia Medica, China Academy of Chinese Medical Sciences, Beijing, 100700 China; 3 Shenzhen Research Institute, Beijing University of Chinese Medicine, Shenzhen, 518118 China; 4 Shenzhen Cell Valley Biopharmaceuticals Co., Ltd., Shenzhen, 518118 China

**Keywords:** Combination therapy, Gallic acid, CD19, CAR-T immunotherapy, B-cell lymphoma

## Abstract

Chimeric antigen receptor T (CAR-T) cell targeting CD19 antigen has achieved exhilarative clinical efficacy in B-cell malignancies. However, challenges still remain for the currently approved anti-CD19 CAR-T therapies, including high recurrence rates, side effects and resistance. Herein, we aim to explore combinatorial therapy by use of anti-CD19 CAR-T immunotherapy and gallic acid (GA, an immunomodulatory natural product) for improving treatment efficacy. We assessed the combinatorial effect of anti-CD19 CAR-T immunotherapy with GA in cell models and a tumor-bearing mice model. Then, the underlying mechanism of GA on CAR-T cells were investigated by integrating network pharmacology, RNA-seq analysis and experimental validation. Furthermore, the potential direct targets of GA on CAR-T cells were explored by integrating molecular docking analysis with surface plasmon resonance (SPR) assay. The results showed that GA significantly enhanced the anti-tumor effects, cytokine production as well as the expansion of anti-CD19 CAR-T cells, which may be mainly through the activation of IL4/JAK3-STAT3 signaling pathway. Furthermore, GA may directly target and activate STAT3, which may, at least in part, contribute to STAT3 activation. Overall, the findings reported here suggested that the combination of anti-CD19 CAR-T immunotherapy with GA would be a promising approach to increase the anti-lymphoma efficacy.

**Supplementary Information:**

The online version contains supplementary material available at 10.1186/s43556-023-00122-6.

## Introduction

Chimeric antigen receptor (CAR) T cell therapy represents an influx of revolutionary strategy for treating relapsed or refractory tumors [1]. CARs are engineered synthetic receptors that redirect T cells to recognize tumor-associated surface antigens in a major histocompatibility complex (MHC)-independent manner, resulting in strong anti-tumor immune response of CAR-T cells [2]. In recent multicenter clinical trials, initially approved anti-CD19 CAR-T therapies have achieved high complete remission rates (70–90%) for pediatric and adult patients with specific B cell malignancies, bringing renewed hope to cancer patients who previously had limited treatment options [3]. Despite the potential of this emerging therapeutic modality, the relapse rate (21–35%) is still high after anti-CD19 CAR-T induced remission，probably due to limited T-cell expansion, T-cell exhaustion as well as escape or down-modulation of CD19 antigen [1, 4–6]. Additionally, anti-CD19 CAR-T therapy may induce serious unfavorable effects, especially cytokine release syndrome, impeding its clinical use [7]. For the above reasons, novel therapeutic approaches are being explored with the goal of optimizing the function and increasing the safety of CAR-T therapy.

Intensive efforts have focused on engineering more powerful CARs to improve the curative effects of CAR-T cells and expand their clinical applications in a wider spectrum of malignancies [2]. For instance, Hu et al. engineered CAR-T cells with dual antigen targeting of CD19 and CD22, which could recognize the two tumor-associated antigens simultaneously and thus showed significant clinical therapeutic efficacy with low CD19^−^ relapse rate [8]. Adachi and co-workers equipped CAR-T cells with co-expressing IL-7 and CCL19 to create a favorable immunological milieu for improving immune cell infiltration and CAR-T cell survival in solid tumors [9]. Based on a study by Carnevale et al., knocking out RASA2, a RAS GTPase-activating protein (RasGAP), in CAR-T cells by use of the CRISPR/Cas9 system promoted CAR-T cell activation, antigen sensitivity, long-term persistence and effector function [10]. Although the anti-tumor effects of CAR-T cells have been significantly improved via genome engineering approaches, there still remain some potential risks, especially off-target mutations and insertional mutagenesis. Thus, it is necessary to develop novel and alternative strategies to prevent or address these issues.

With the growing therapeutic arsenal in oncology, rational combination of CAR-T therapy with other types of anticancer therapies has emerged as one of the most promising therapeutic avenues against cancer [11]. Recently, a combination of CAR-T therapy together with chemotherapeutic agents has drawn increasing attention and has been widely studied in preclinical or clinical investigations [12]. For instance, Wang et al. suggested decitabine-treated CAR-T cells displayed persistent antitumor activities, which may be via decitabine-mediated epigenetic reprograming [13]. Xu et al. found that STING agonist could promote CAR-T cell trafficking in breast cancer [14]. Fraietta and co-workers found that ibrutinib increased the proliferative capacities of anti-CD19 CAR-T by decreasing immunosuppressive PD-1 (programmed cell death 1) and CD200 expression on T cells and tumor cells, respectively [15]. Moreover, several conventional chemotherapeutic drugs, such as doxorubicin, fluorouracil and cyclophosphamide, have been report to specifically control the immunosuppressive functions of regulatory T-cells and/or myeloid-derived suppressor cells, resulting in enhanced antitumor immunity [16]. These studies suggest that chemotherapeutic agents can improve immune function while reducing tumor burden, which hold promise to be effective tools for amplifying the efficacy of CAR-T therapy. However, most of these chemotherapeutic drugs have serious adverse effects which have restricted their widespread application [17, 18]. Hence, there continues to be a great need for novel and safe combinatorial treatment approaches for CAR-T immunotherapy to maximize their therapeutic potential.

Gallic acid (GA) is an immunomodulatory natural product existed in various fruits, vegetables and traditional Chinese medicines, such as *Rhus chinensis* Mill. and *Punica granatum* L, which has the advantages of low cost and high safety [19]. It has been suggested that GA may stimulate the proliferation and enhance the activity of natural killer cells [20]. Gallic acid and 1-methyl-d-tryptophan (an indoleamine-2,3-dioxygenase inhibitor) cross-linked small molecule could significantly suppress tyrosinase expression and modulate the ratio of CD4^+^, CD8^+^, and regulatory T cells (Treg cells) in melanomas, yielding a remarkable anti-tumor effect *in vivo* [21]. In addition, GA could significantly augment the anti-leukemic efficacy of chemotherapeutic drugs, such as daunorubicin, cytarabine and asparaginase [19, 22]. Accordingly, considering GA’s positive role in immune regulation and the enhanced efficacy of traditional chemotherapeutic drugs when co-administration with GA, we hypothesized that combination therapy incorporating GA and CAR-T cells may have huge prospects for improving the function of CAR-T cells and achieving a multiplier effect. Hence, in this study, we aimed to investigate the *in vitro* and *in vivo* anti-lymphoma efficacy of anti-CD19 CAR-T therapy in combination with GA. The effects and the underlying mechanisms of GA on anti-CD19 CAR-T cells were also elucidated. Our study may provide a scientific basis for the clinical potential of combining anti-CD19 CAR-T therapy with GA against relapsed/refractory B-cell lymphoma.

## Results

### GA improves the viability and proliferation of CAR-T cells

**Fig. 1:**
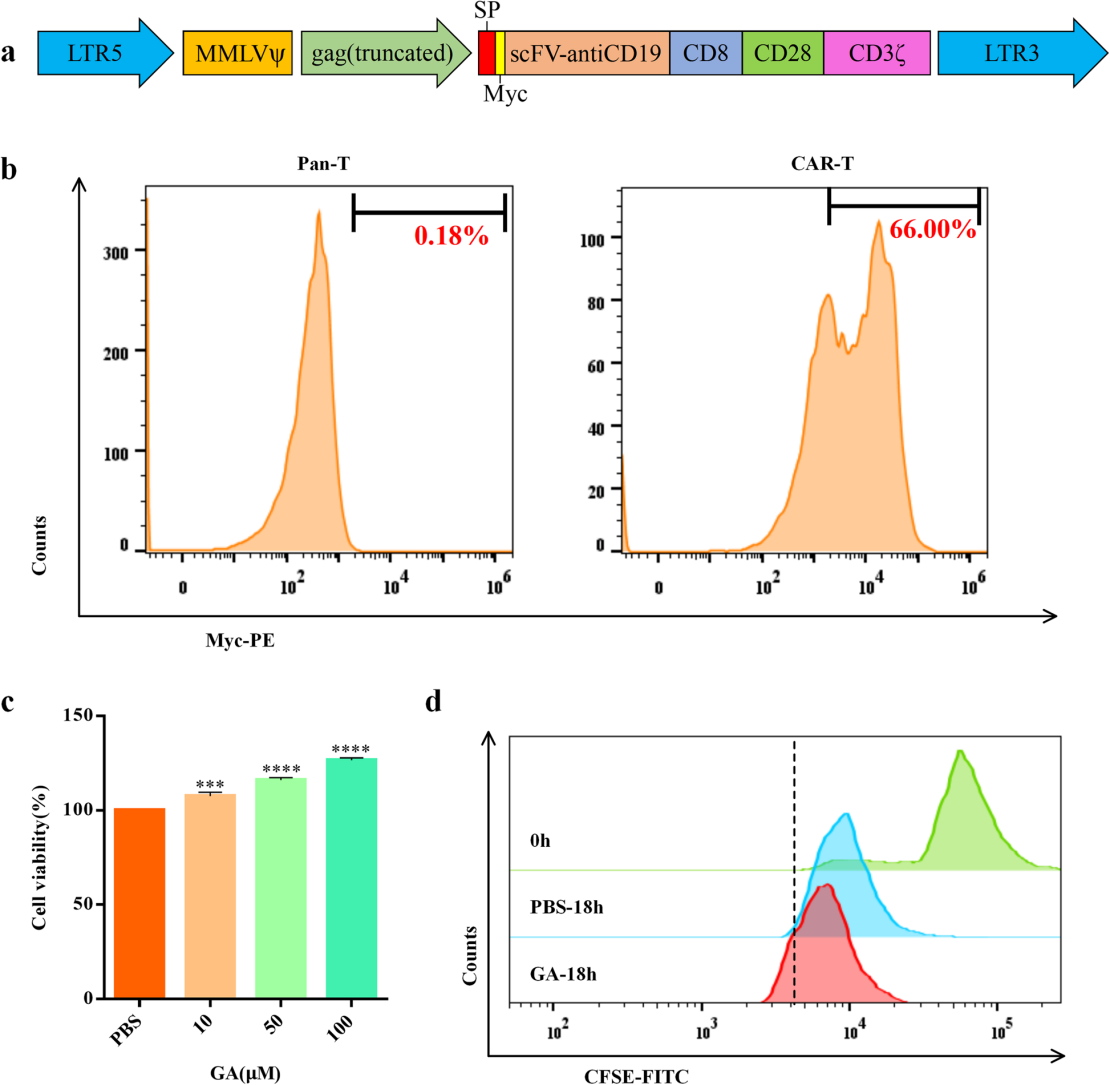
GA improved the cell viability and proliferation of anti-CD19 CAR-T cells *in vitro*. **a** The construction of anti-CD19 CAR. **b** The transduction efficiency of CD19 CAR in primary T cells. **c** The cell viability of anti-CD19 CAR-T cells determined by PrestoBlue™ assay. Values were expressed as the means ± S.D.. *P < 0.05; **P < 0.005; ****P < 0.0001 (*n* = 6). **d** The proliferation of anti-CD19 CAR-T cells determined by CFSE assay (GA: 100 μM). PBS, phosphate buffered saline; FITC, fluorescein isothiocyanate

CAR-T cells were successfully constructed by transducing PBMCs with retroviral vectors encoding the CD19/CD8-CD28-CD3ζ transgene (Fig. 1a), which was confirmed by fluorescence-activated cell sorting (FACS) analysis using phycoerythrin (PE)-conjugated anti-myc antibody (Fig. 1b). Then, we investigated the impact of GA on CAR-T viability through PrestoBlue™ assay at 10, 50 and 100 μM. We found that GA dose-dependently increased viability of CAR-T cells (Fig. 1c). For further validating the aforementioned results, we next assessed the impact of GA on CAR-T proliferation through monitoring carboxyfluorescein diacetate succinimidyl ester (CFSE) dilution. As shown in Fig. 1d, treatment with GA at 100 μM for 18 h could slightly promote the proliferation of CAR-T cells. Collectively, these findings suggested GA may potentiate CAR-T cell activity.

### GA augments the cytotoxic function of anti-CD19 CAR-T *in vitro*

**Fig. 2:**
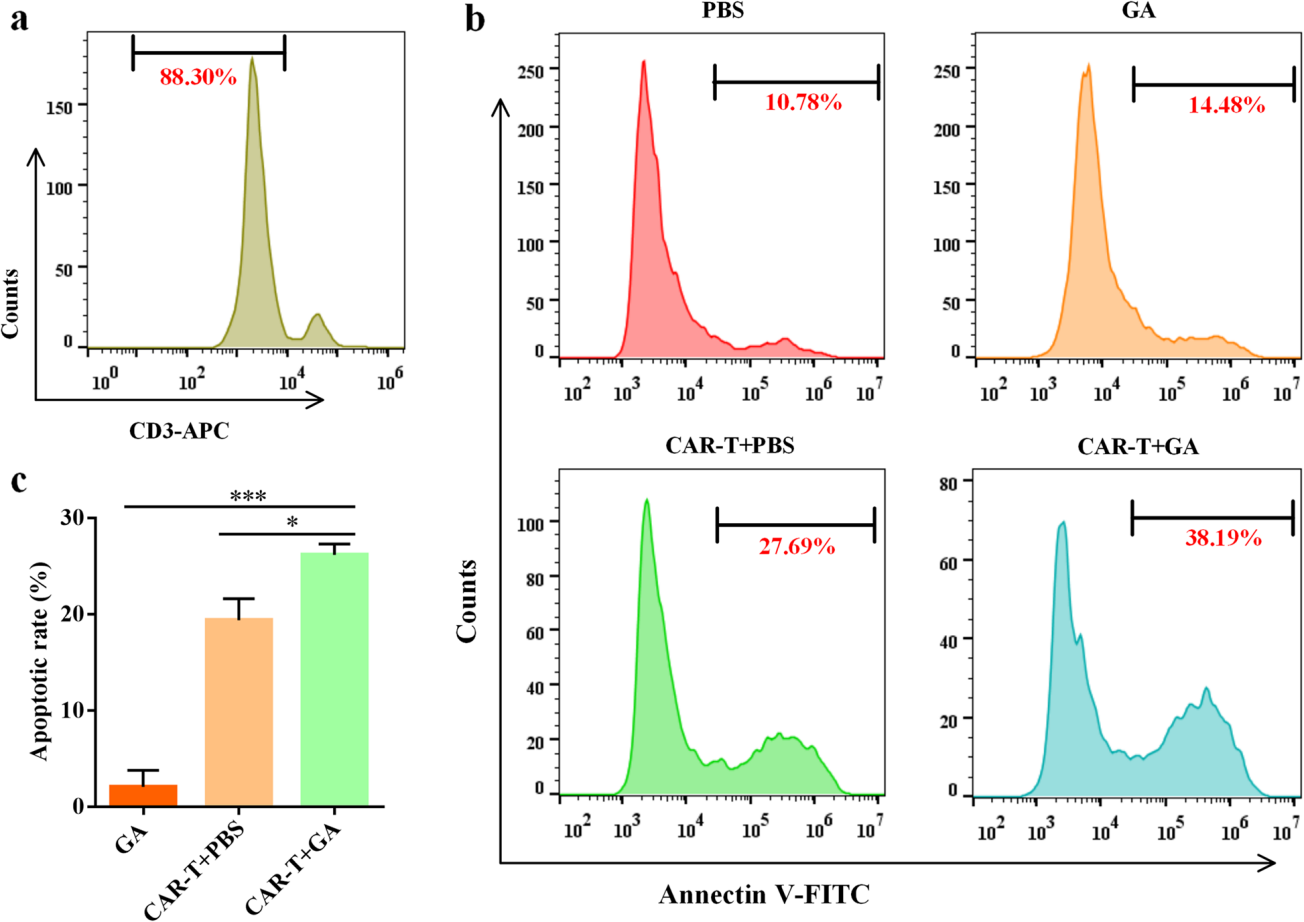
GA enhanced the cytotoxic function of anti-CD19 CAR-T cells *in vitro.***a** Representative FACS plot for identifying Raji cells (CD3-negative cells). **b** Representative FACS analysis of the apoptosis of Raji cells. **c** The apoptotic rate of Raji cells after normalized to no CAR-T cell/GA wells (PBS group). *P < 0.05; ***P < 0.005 (*n* = 3). The concentration of GA was 100 μM and the effector: target ratio was 1:8

The effect of GA on CAR-T cell cytotoxicity was investigated by FACS apoptosis assay after cells being stained with allophycocyanin (APC)-conjugated anti-human CD3 antibody and fluorescein isothiocyanate (FITC)-conjugated Annexin V. As shown in Fig. 2a, cells were fractionated by FACS based on the fluorescence signal into CD3-positive (T cell) and CD3-negative cells (Raji cell). Then, CD3-negative cells were gated out to detect the apoptotic rate of Raji cells (The proportion of Annexin V- positive cells). As shown in Fig. 2b and c, combination of GA (100 μM) with CAR-T cell significantly induced more apoptosis of Raji cells than GA or CAR-T alone. Interestingly, we noted that the sum of the apoptotic rate of CAR-T+ phosphate buffered saline (PBS) group (at the effector/target ratio of 1:8) and GA group was less than that of CAR-T + GA group after normalized to no CAR-T cell/GA wells (PBS group). These findings indicated that GA could enhance the cytotoxic function of anti-CD 19 CAR-T cells against Raji cells *in vitro*.

### Enhanced anti-lymphoma effects of anti-CD19 CAR-T combined with GA *in vivo*

**Fig. 3:**
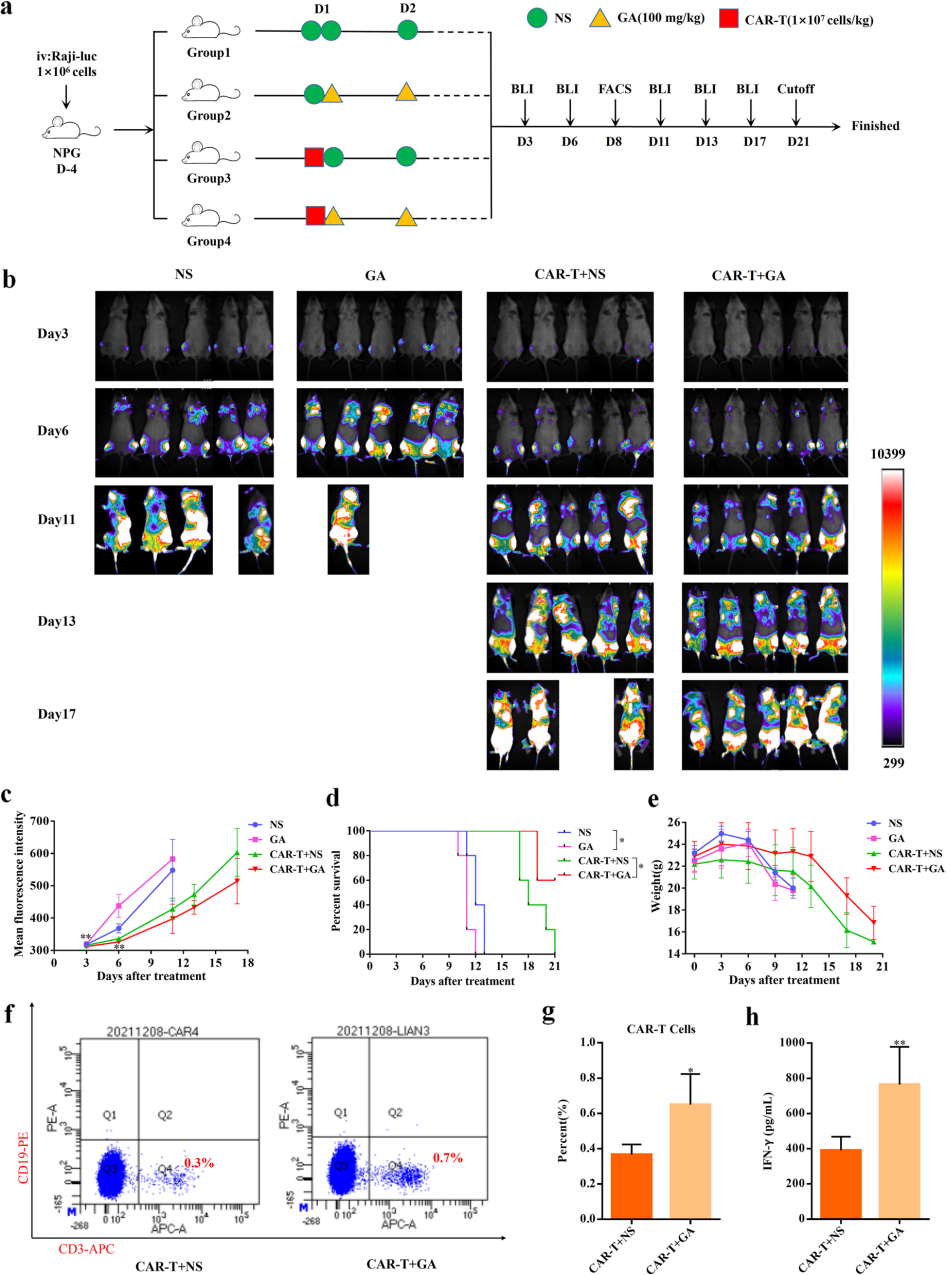
GA enhanced anti-lymphoma activity of anti-CD19 CAR-T cells *in vivo.***a** The schema of the experimental design for this study. **b** Tumor burden in mice monitored by BLI and **c** presented as the mean fluorescence intensity. **P < 0.01 (*n* = 5), compared CAR-T + NS group with CAR-T + GA group at the same time point. **d** Survival analysis of mice after CAR-T and/or GA treatment. *P < 0.05 (*n* = 5). **e** Body weight variation of mice after CAR-T and/or GA treatment (*n* = 5). **f** Representative FACS analysis of CAR-T and Raji cells in mice blood.; **g** The percent of CAR-T cells in mice blood detected by FACS. *P < 0.05 (*n* = 5) **h** The IFN-γ level in mice blood quantitated by ELISA. **P < 0.01 (*n* = 5). NS, normal saline; D, day

To further confirm our *in vitro* findings, the *in vivo* anti-lymphoma effects of the combination of anti-CD19 CAR-T with GA were also evaluated. For this purpose, a Raji-Luc cell-based lymphoma model was developed in the severe immunodeficient NOD-Prkdc^scid^ Il2rg^tm1^/Vst (NPG) mice and the experimental design was presented in Fig. 3a. Mice were treated with normal saline (NS), GA, CAR-T + NS, or CAR-T + GA. Bioluminescence imaging (BLI) revealed that NPG mice exhibited slower progression of tumor growth in the combination group as compared with that in CAR-T + NS group or GA group (Fig. 3b and c). Consistent with the above findings, the survival rate (Fig. 3d) for the combination group was higher than that of CAR-T + NS or GA group. And mice in CAR-T + GA group exhibited less body weight variation than their counterparts in CAR-T + NS group (Fig. 3e). On day 8, tail blood was collected, and then CAR-T and Raji cells in mouse blood were detected by FACS. Significantly higher proportions of human T cells (Fig. 3f and g) were observed in the combination group than those in CAR-T + NS group. However, the Raji cells could not be detected in both groups by FACS, which may be due to the extremely low level of Raji cells in mouse blood. In addition, we measured the human IFN-γ (an important functional cytokine related to T-cell activities) production in mouse blood by enzyme linked immunosorbent assay (ELISA), and found that CAR-T + GA group had significantly higher IFN-γ level than CAR-T + NS group (Fig. 3h). All these findings demonstrated that GA could improve the proliferation and antitumor ability of CAR-T *in vivo*.

### The action mechanism of GA on T cell proliferation via network pharmacology

**Fig. 4:**
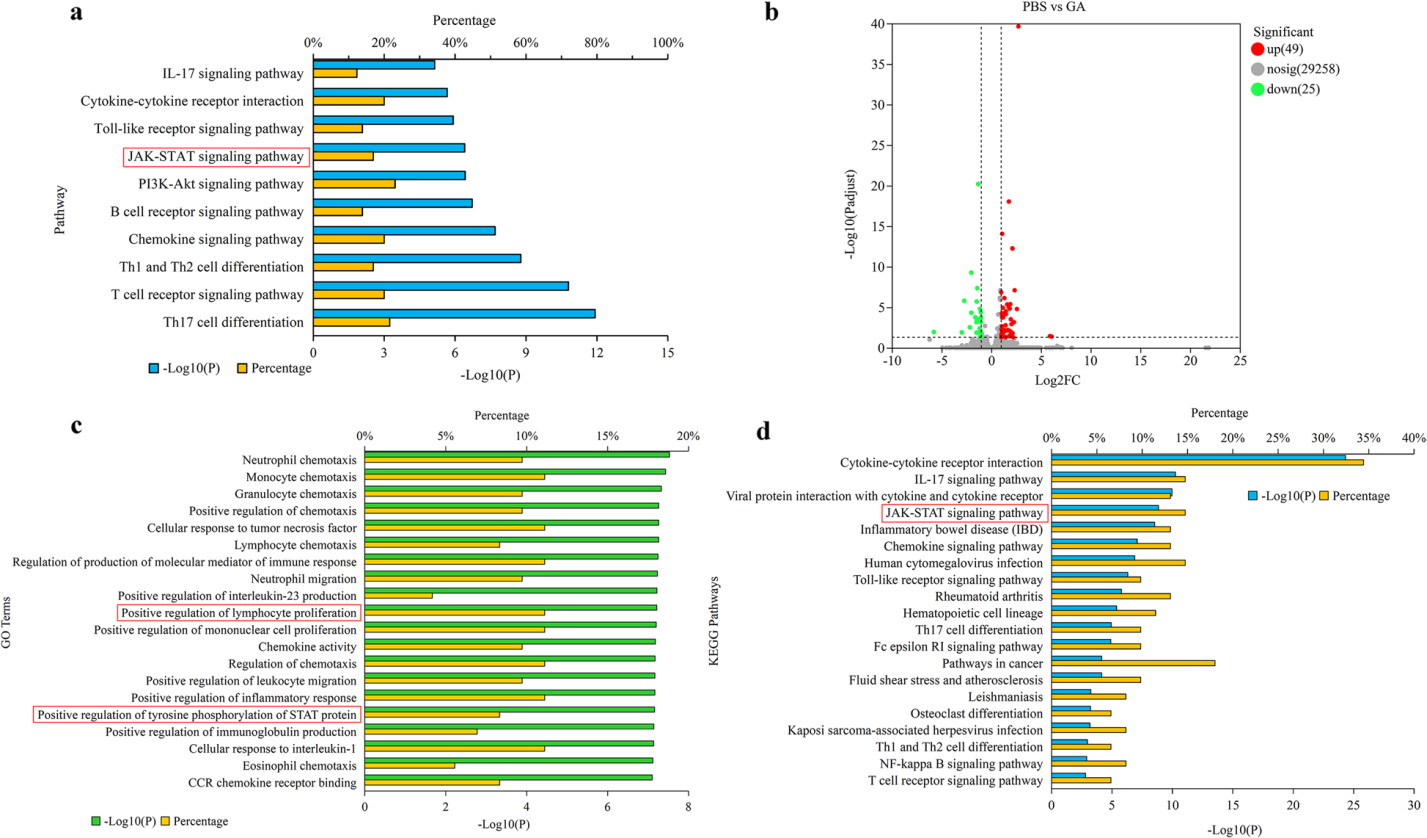
The action mechanism of GA on CAR-T. **a** Pathway enrichment analysis of key hubs from network pharmacology analysis. **b** The volcano plot of DEGs from RNA-seq analysis (GA:100 μM; *n* = 3). **c** The top 20 significantly enriched GO terms by RNA-seq analysis. **d** The top 20 significantly enriched KEGG pathways by RNA-seq analysis. (**a c d**) The ordinate stands for the main pathways, the primary abscissa stands for minus log 10(*P*), and the secondary abscissa stands for the percentage of key hubs or DEGs involved in the corresponding main pathways out of total key hubs or DEGs

The pharmacological mechanism of GA on T cell proliferation (TP) were preliminarily investigated by network pharmacology analysis. As given in supplementary tables, 98 GA-related targets (Supplementary Table 1) were predicted by MedChem Studio and 141 TP-related targets (Supplementary Table 2) were obtained from Online Mendelian Inheritance in Man (OMIM) database. To scientifically unveil the relationships between GA and TP, the “GA-related targets-TP-related targets” interaction network was built, and 1482 edges (Supplementary Table 3), representing the most influential interactions within a network, were identified. The hub subnetwork was further constructed, and 66 key hubs (Supplementary Table 4) were chosen as the core targets based on the values of the network topological parameters (Degree Centrality (DC) ≥ 9, Betweenness Centrality (BC) ≥ 0.001456, and Closeness Centrality (CC) ≥ 0.394892). Functionally, these key targets were highly enriched in multiple Kyoto Encyclopedia of Genes and Genomes (KEGG) pathways (Top 5) including IL-17, cytokine-cytokine receptor interaction, Toll-like receptor, JAK-STAT and PI3K-Akt signaling pathways (Fig. 4a).

### Transcriptional changes of CAR-T after treatment with GA

**Fig. 5:**
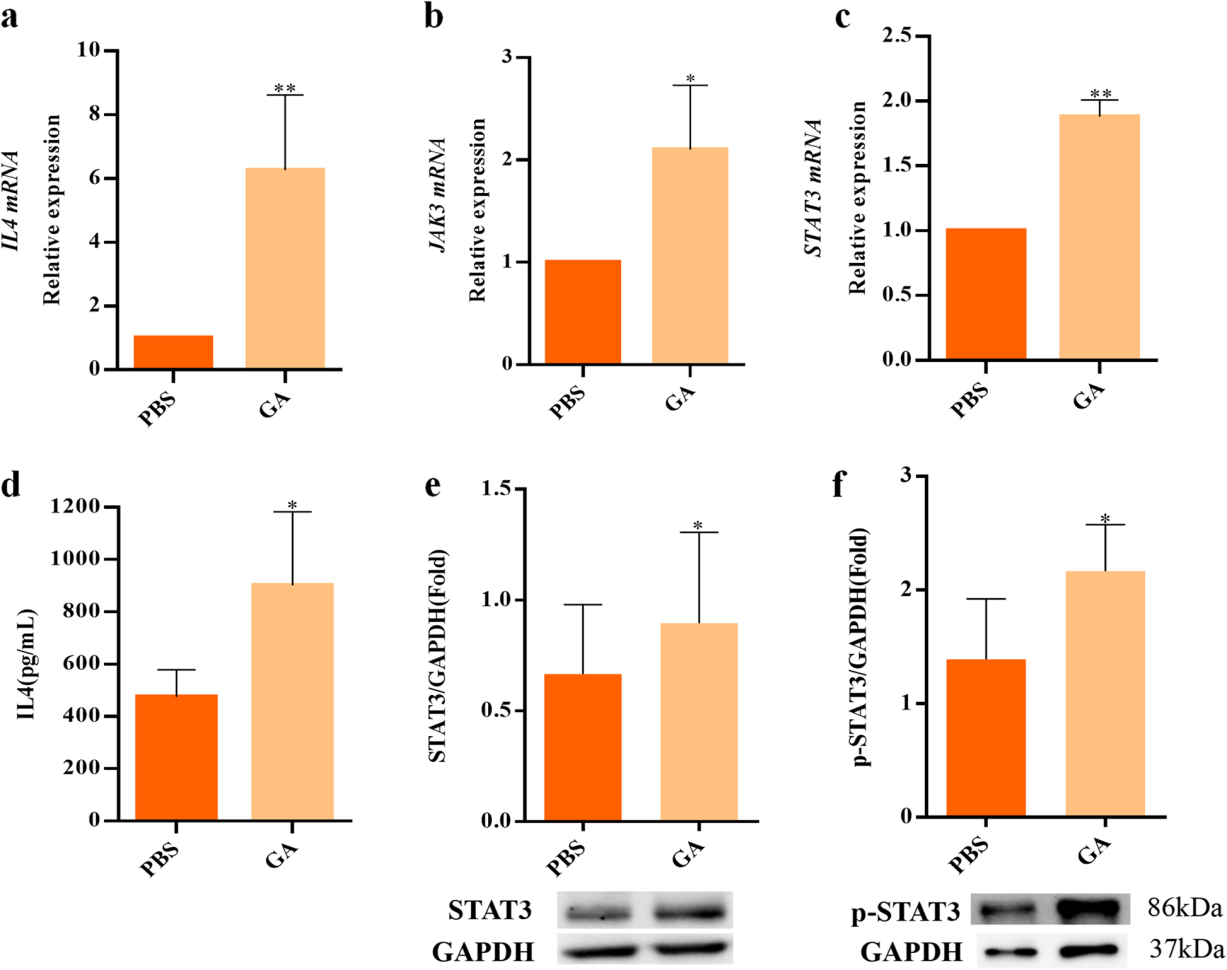
Verification of the major differentially expressed genes and proteins in IL4/JAK3-STAT3 pathway. RT-qPCR results showed the increased expression levels of **a** IL4, **b** JAK3 and **c** STAT3 in GA (100 μM) group. **d** ELISA results showed the increased level of IL4 in GA (100 μM) group. Western blotting assay showed the increased levels of **e** STAT3 and **f** p-STAT3 in GA (100 μM) group. *P < 0.05; **P < 0.01 (*n* = 3)

To further illustrate the underlying mechanisms of GA on anti-CD19 CAR-T, we conducted RNA-seq analysis of CAR-T (with or without GA treatment) in a “resting state” (not stimulated by tumor cells). After quality control, 74 transcripts (Fig. 4b) were differentially expressed between the two groups (49 upregulated and 25 downregulated). Next, to investigate the functional associations of these differentially expressed genes (DEGs), the most significant pathways (Top 20) were enriched by Gene Ontology (GO) and KEGG analysis (Fig. 4c and Fig. 4d). Interestingly, through GO analysis, we found positive regulation of lymphocyte proliferation (GO:0032946), mononuclear cell proliferation (GO:0050920) and tyrosine phosphorylation of STAT protein (GO:0002639) were the highly significant enriched ontologies. As can be seen from KEGG analysis, JAK-STAT signaling was also highly enriched (Top 5), consisting with the results of network pharmacology analysis. Additionally, previous studies have demonstrated that antigen-dependent JAK-STAT3/5 pathway activation could facilitate the expansion and enhance antitumor effects of CAR-T [23]. Based on these findings, we deduced that GA may promote CAR-T cell proliferation mainly through JAK-STAT signaling pathway. After further analyzing the DEGs from RNA-seq analysis, we verified the expression level of major genes and proteins associated with JAK-STAT pathway using quantitative real-time polymerase chain reaction (qRT-PCR), ELISA and western blot assay. The results (Fig. 5) indicated that activation of IL4/JAK3-STAT3 signaling pathway may be a mechanism of action of GA.

### The interaction between GA and STAT3

**Fig. 6:**
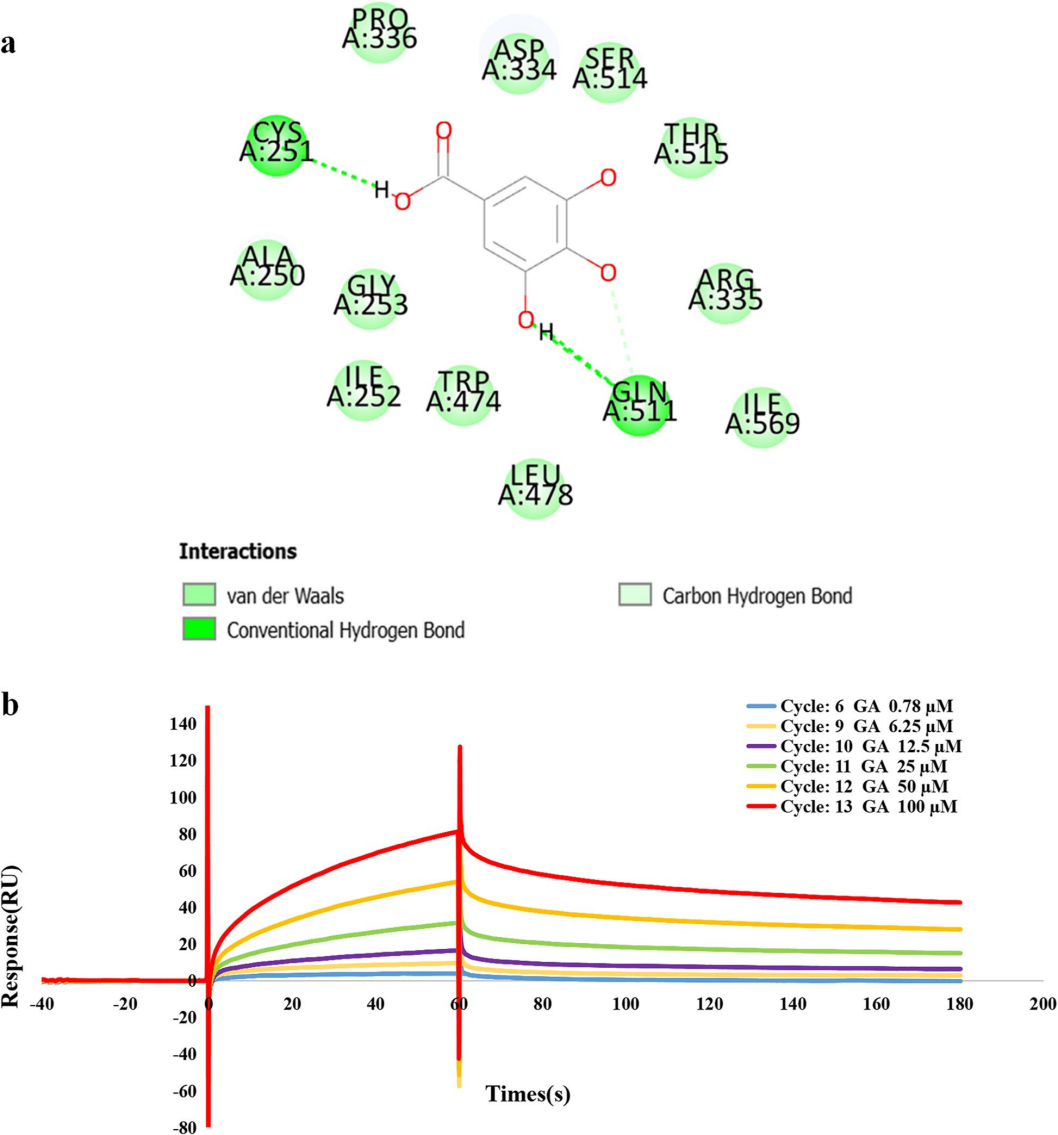
The interaction between GA and STAT3. **a** Molecular docking analysis; **b** SPR analysis

Considering the important role of STAT3 activation in CAR-T cell efficiency [24, 25], the interaction between GA and STAT3 was attempted to investigate by molecular docking and surface plasmon resonance analysis (SPR). As shown in Fig. 6a, GA formed two hydrogen bonds with CYS251 and GLN511, and the corresponding docking score was 80.3427. SPR analysis (Fig. 6b) also demonstrated that GA-STAT3 showed good binding affinity (Ka (the association rate constant) = 194.3 M^− 1^ s^− 1^；Kd (the dissociation rate constant) = 4.19E-03 s^− 1^; KD (affinity constant) = 21.6 μM). Taken together, these results indicated that targeting STAT3 by GA may be one of the important mechanisms involved in the activation of STAT3-mediated signaling, leading to enhanced CAR-T cell activity.

## Discussion

Although several clinical successes of anti-CD19 CAR-T therapy have been acknowledged, how to improve and maintain the efficacy of CAR-T is a central question currently facing the field [26]. Multiple studies have demonstrated that small molecules can improve the efficacy of CAR-T cells, inhibit suppressive immune cells and enhance the antigenicity of cancer cells [16]. Thus, concurrent administration of small molecules and CAR-T cells may achieve more prominent curative effects than monotherapy. More recently, the application of plant-derived immunomodulators (PDIs) as adjunctive therapies has become a promising strategy against cancer. Accumulating evidence suggests that PDIs combined with chemotherapeutic drugs enhance the therapeutic efficiency and reduces the adverse effects of chemotherapy, which improves the quality of life of patients [27]. However, so far, there were very few studies about the combination of PDIs with CAR-T cells for combating cancer [28].

In this study, we demonstrated that GA could enhance the antitumor efficiency of anti-CD19 CAR-T both *in vitro* and *in vivo*. Due to the relative low dose of CAR-T used in this study, the xenograft tumors in CAR-T + GA and CAR-T + NS groups were not be completely eliminated. It is still necessary to pinpoint the doses and interactions between CAR-T cells and GA. Interestingly, GA alone may slightly promote the progression of Raji cells compared with the NS group (Fig. 3b-d), which in turn suggested that GA combats tumors through direct effects on T cells but not on Raji cells. In addition, Liu et al. [29] evaluated the curative effect of ibrutinib combined with anti-CD19 CAR-T in both subcutaneous and tail vein tumorigenic mice (Raji cells). The result showed that the synergistic effect could only be observed in the subcutaneous tumorigenic model, which may be due in part to the differences in the complex tumor microenvironment between the two models. Similarly, GA combined with anti-CD19 CAR-T may have a more significant therapeutic effect in subcutaneous tumorigenic mouse model, which is definitely worth further exploration in future.

We further explore the action mechanisms of GA on CAR-T cells by network pharmacology and transcriptomic analysis. Interestingly, most highly enriched pathways by network pharmacology analysis, such as JAK-STAT, IL-17 signaling, cytokine-cytokine receptor interaction, Toll-like receptor, chemokine signaling, Th1 and Th2 cell differentiation, T cell receptor signaling and Th17 cell differentiation, could also be found in KEGG enrichment results of the transcriptional data, demonstrating the predictive accuracy of network pharmacology analysis. Among them, up-regulation of IL4/JAK3-STAT3 signaling pathway has been validated to play a critical role in GA acting on CAR-T cells. It is well known that T cell proliferation is largely relied on the binding of IL-2 and IL-4 to cell surface receptors. IL-4 can stimulate the rapid activation of JAK3, thereby inducing STAT3 and STAT5 phosphorylation and nuclear migration [30, 31], which results in enhanced T cell activity [23, 32, 33]. Besides, investigation of other regulation mechanisms, especially IL-17 signaling pathway and cytokine-cytokine receptor interaction, is still needed to gain a comprehensive view of GA acting on CAR-T cells.

STAT3 activation is considered as a remarkable index of CAR-T cell potency. For instance, Fraietta et al. found that anti-CD19 CAR-T cells from complete-responding patients showed higher expression levels of STAT3 signaling mediators and targets than those from non-responders, which facilitated the proliferation of CAR-T cells [24]. In line with this, Kagoya et al. designed a novel CAR construct capable of activating STAT3-mediated signaling, leading to superior antitumor effects of anti-CD19 CAR-T cells [23]. Similarly, CAR-T cells expressing IL-4/21 receptors promoted STAT3 phosphorylation, eventually promoting Th17-like polarization and enhancing CAR-T cell potency [34]. Therefore, considering our current findings and those of previous studies, it is clear that STAT3 activation plays an essential role in the potentiating effect of GA on anti-CD19 CAR-T efficiency. We then tried to test the interactions between GA and STAT3 by molecular docking and SPR analysis, and it was interesting to find out that GA could directly target STAT3, which may, at least in part, contribute to STAT3 activation. To fully elucidate the regulation mechanisms of GA in STAT3-mediated signaling, the downstream elements of STAT3, such as the stability of STAT3, the DNA binding ability and the target gene expression of STAT3, need to be explored in future studies.

Various barriers restrict the efficacy of CAR-T therapy, including insufficient antitumor immunity, antigen escape, suboptimal persistence, restricted trafficking as well as limited tumor infiltration [35]. Thus, in addition to the enhanced CAR-T cell expansion and cytotoxicity, more aspects, such as exhaustion, apoptosis and subsets of CAR-T cells, are proposed to be investigated to fully reflect the spectrum of CAR-T cell efficiency when co-administration with GA. And it is also suggested to test the above mentioned functions of GA-pretreated CAR-T cells (during ex vivo expansion) in the *in vivo* model, which may broaden the therapeutic benefits of GA and greatly contribute to the preclinical evaluation of the combination of anti-CD19 CAR-T cells and GA in the treatment of B-cell malignancies.

Taken together, this work afforded evidence that the combination with GA or other herbal derivatives could augment the treatment efficacy of CAR-T immunotherapy. When CAR-T is in combination with GA, we may consider reducing the dose of CAR-T appropriately for alleviating the risk of cytokine storm. In addition, GA may serve as a lead compound for developing synthetic analogues with improved bioactivity. Even though the aforementioned results are promising, some deficiencies of this work remain to be improved and further studies are demanded to draw more definitive conclusions about the potentiation of CAR-T efficacy by GA treatment.

## Material and methods

### Cell lines and reagents

Raji cells were obtained from the American Type Culture Collection (ATCC, Manassas, VA, USA) and Raji cells stably expressing firefly luciferase (Raji-Luc) were purchased from Beijing Vitalstar Biotechnology Co., Ltd.. Both cell lines were cultured in RPMI-1640 medium (Gibco) supplemented with 10% fetal bovine serum (Gibco) and 100 U/mL penicillin/streptomycin (Invitrogen). Gallic acid was provided as a powder (purity ≥98%, Vetec™), and dissolved in normal saline before use.

### Manufacturing of anti-CD19 CAR-T cells

The CD19-CAR retrovirus was kindly provided from Professor Jianxun Wang. CAR consisted of an extracellular single chain variable fragment (scFv) specific for CD19, followed by a CD8 hinge-transmembrane domain, CD28 costimulatory domain and CD3z intracellular signaling domain. The CAR-T cells were generated by retrovirus infection. In detail, after informed consent was obtained, whole blood samples derived from healthy donors were collected for isolating peripheral blood mononuclear cells (PBMCs) by Ficoll density gradient centrifugation (Lymphoprep, Stemcell, Canada). Then, OKT3 (soluble anti-human CD3 antibody) and IL-2 obtained from Sino Biological were added into the AIM-V medium (Gibco, USA) for the activation of PBMCs at the final concentrations of 100 ng/mL and 100 U/mL, respectively. 48 h later, activated T cells were infected with retroviral vectors carrying CD19 CAR by use of the spin inoculation method [36]. Finally, The CAR-T cells were expanded and the transduction efficiency was evaluated by flow cytometric analysis (CytoFLEX, Beckman Coulter, USA). Briefly, CAR-T cells were stained with PE-conjugated anti-myc (Invitrogen, USA) antibody, and FACS data was analyzed by CytoFLEX Software (Beckman Coulter, USA) and FlowJo (Version 10.0.7).

### Cytotoxicity assay

Raji cells (1.6 × 10^5^ /well) were divided into three treatment groups, given CAR-T cells (2 × 10^4^ /well) with or without GA (100 μM), GA (100 μM) and the control (PBS) group (*n* = 3). After incubation for 12 hours, the cells were stained with APC-conjugated anti-human CD3 antibody (BD Pharmingen, USA) and FITC-conjugated Annexin V (Gene-Protein Link, China) for half an hour. The apoptosis rate of Raji cells was then measured by flow cytometric analysis (CytoFLEX, Beckman Coulter, USA), and FACS data was analyzed by CytoFLEX Software (Beckman Coulter, USA) and FlowJo (Version 10.0.7).

### Cell viability assay

CAR-T cells (4 × 10^4^ /well) were treated with GA at 10, 50, 100 μM for 24 h. Cell viability was evaluated using PrestoBlue™ reagent (Invitrogen, USA) and the fluorescence intensity (excitation/emission: 560 nm/590 nm) of each well was determined by a microplate reader (Molecular Devices, USA). Relative cell viability was calculated following normalization to untreated control cells.

### Cell proliferation assay

CAR-T cells were pre-labeled with CFSE (BD, USA) according to the provided protocol. Then, the labeled cells were treated with GA at 100 μM for 18 h. Finally, the cell division was measured by monitoring the corresponding decrease in cell fluorescence via flow cytometry (BD LSRFortessa, USA), with the data processed by FlowJo (Version 10.0.7).

### Mice studies

NPG mice (Beijing Vitalstar Biotechnology Co.,Ltd.) were used for the establishment of a mouse xenograft model. Mice (6–8 weeks old) were injected intravenously (i.v.) with Raji-Luc cells (1 × 10^6^ cells/mouse). 4 days later, these animals were randomly assigned to three treatment groups (*n* = 5), given CAR-T (1 × 10^7^ /kg) with or without GA (100 mg/kg for 20 days), GA (100 mg/kg for 20 days) and the control (*n* = 5). In this research, CAR-T and GA were administered by intravenous (i.v.) and intraperitoneal (i.p.) injections, respectively.

From the 3rd day after CAR-T inoculation, mice were monitored by a multifunctional *in vivo* imaging device (MIIS, Molecular Devices, USA) twice a week. Before BLI, animals were intraperitoneal injection with VivoGlo™ Luciferin (150 mg/kg, Promega, USA). 3 min later, the mice were imaged under isoflurane anesthesia using a 5 min exposure time. The mean fluorescence density was calculated by MetaMorph software (Molecular Devices, USA).

On the 8th day, the blood of mouse was collected from tail veins. Then, the cells were stained with APC-conjugated anti-CD3 (BD, USA) antibody and PE-conjugated anti-CD19 antibody (BD, USA) for 2 h. After treatment with erythrocyte lysis solution (Beyotime Biotechnology, Shanghai, China), the cells were washed and then stained with 7-AAD (5 μL/test, Biolegend, USA) for 10 min before flow cytometry analysis (BD LSRFortessa, USA). On the 20th day, plasma IFN-γ level was quantified by an ELISA assay (Proteintech, China). All mice died of natural causes and survival curves were recorded.

### Cytokine measurements

The levels of IFN-γ and IL4 from mouse plasma and cell culture supernatant were determined by ELISA following the manufacturer’s protocols (Proteintech, China).

### Network pharmacology analysis

Network pharmacology analysis was carried out according to our previous studies [37, 38]. Briefly, the GA-related targets were searched by MedChem Studio 3.0 (Simulations Plus, USA), setting confidence score at 0.6. And the targets related to T cell proliferation were retrieved from OMIM database, using “T cell proliferation” as the query. Then the protein-protein interaction (PPI) network of GA-related targets and TP-related targets was built by use of STRING database, setting the confidence score at 0.4 and the species limited to *Homo sapiens*. Furthermore, the hub subnetwork was built using the obtained PPI data and visualized by Cytoscape 3.7.0. Next, we calculated the values of DC, BC and CC for assessing the topological significance of hub nodes. The medians for the above topological parameters were used as the screening criteria to acquire the key hubs. Finally, to uncover the potential functions of these key hubs, KEGG pathway analysis was conducted by use of the Database for Annotation, Visualization, and Integrated Discovery (DAVID) system. We considered *P* values below 0.05 as statistically significant.

### RNA-sequencing and bioinformatics analysis

Total RNA extraction from CAR-T cells was conducted by use of TRIZOL reagent (Invitrogen, USA) following the provided protocol. The RNA quantity and quality were assessed using a SpectraMax Spectrophotometer (Molecular Devices, USA) and a 2100 Bioanalyzer (Agilent, USA), respectively. The construction of sequencing libraries was performed using TruSeq™ RNA sample prep Kit (Illumina, USA) following the provided instructions. Then the constructed libraries were sequenced by a NovaSeq 6000 instrument (Illumina, USA) and 125 bp/150 bp paired-end reads were generated. The transcriptional analysis was performed by Shanghai Majorbio BioPharm Technology Co., Ltd. And the DESeq2 package was used to identify significantly DEGs (*P* < 0.05 and fold-Change > 2). Then, pathway enrichment analysis of DEGs including GO and KEGG were conducted to define functions and pathways altered in CAR-T cells.

### qRT-PCR assay

Quantitative confirmation of the selected DEGs was performed by qRT-PCR assay. After reversely transcription using a cDNA Synthesis Kit (Invitrogen, USA), the cDNA of the total mRNA was obtained. Then, the quantitative PCR reaction using SYBR® Green Premix *Pro Taq* HS qPCR Kit (Accurate Biotechnology, China) was performed on QuantStudio6 Flex system (Applied Biosystems). The gene expression was calculated with the 2^−ΔΔCT^ approach and expressed as the relative fold-change normalized against GAPDH. Table 1 listed the primers used in qRT-PCR.

**Table 1:** Primers used in qRT-PCR

Gene	Primers	Amplicon size (bp)
IL4	Forward: CCAACTGCTTCCCCCTCTG	150
	Reverse: TCTGTTACGGTCAACTCGGTG	
JAK3	Forward: TTCGGGCTACGCAAGGATTTG	143
	Reverse: AGGCTGAGACACTCACCCT	
STAT3	Forward: CATCCTGAAGCTGACCCAGG	225
	Reverse: TCCTCACATGGGGGAGGTAG	
GAPDH	Forward: CAAATTCCATGGCACCGTCA	132
	Reverse: GACTCCACGACGTACTCAGC	

### Western blotting

Total proteins of CAR-T cells were extracted and then quantitated by BCA Protein Assay Kit (Beyotime Biotechnology, China) following the provided protocol. An equal amount of total proteins was electrophoresed on an 8–12% SDS-PAGE gel followed by standard immunoblotting with anti-Stat3 (124H6), or anti-phospho-Stat3 (Tyr705), or anti-GAPDH(D4C6R) antibodies (Cell Signaling Technology, Beverly, USA). The membranes were visualized using chemiluminescent detection reagents (NCM Biotech, Soochow, China) and imaged using the Imaging System (Bio-Rad, USA). Signals were analyzed using ImageJ software.

### Molecular docking stimulation

Molecular docking study was conducted to investigate the interaction between GA and STAT3 using LibDock in Discovery Studio. The structure of STAT3 with higher resolution was obtained from the RCSB protein database and decorated by removing the co-crystallized ligand and water as well as adding polar hydrogens. The three-dimensional structure of GA was generated using Chem3D Pro 12.0. The docking score was applied to assess the binding affinity of GA-STAT3. A binding mode with the highest docking score was chosen and visualized by Discovery Studio. The rest parameters were set to default values.

### Surface plasmon resonance analysis

The SPR assay was performed on a Biacore T200 SPR instrument (GE Healthcare, Sweden) according to manufacturer’s protocols. Briefly, the CM5 sensor chip (Cytiva™, Sweden) was esterified using sulpho-*N*-hydroxysuccinimide (NHS)/1-ethyl-3-(3-dimethylaminopropyl) carbodiimide (EDC) cross-linking reaction at a pH of 4.5. Then, STAT3 was conjugated onto the surface of the chip with immobilization level of 14,860 response unit. GA was serially diluted with a running buffer containing 5% DMSO from 0.78 to 100 μM, and was injected into the STAT3 protein channel and the blank channel (negative control), respectively, at 10 μL/min. Biacore T200 analysis software was employed for fitting the SPR curves according to the steady-state affinity model (1:1), and the kinetics (Ka and Kd) and affinity constants (KD) were calculated.

### Statistical analysis

Statistical analyses were conducted by use of Prism GraphPad 8 and expressed as mean ± S.D. (*n* ≥ 3). Two-group comparison was conducted by Student’s t-test. And a log-rank Mantel-Cox test was employed for comparing survival differences. We considered *P* values below 0.05 as statistically significant.

## Supplementary Information

**Additional file 1: Supplemental Table 1.** GA-related targets. **Supplemental Table 2.** TP-related targets. **Supplemental Table 3.** Interactions between GA-related targets and TP-related targets. **Supplemental Table 4.** Information about key hubs.

## Data Availability

The datasets used and/or analyzed during the current study are available from the corresponding author on reasonable request.

## Abbreviations

CAR-T: Chimeric antigen receptor T
GA: Gallic acid
SPR: Surface plasmon resonance
MHC: Major histocompatibility complex
CRS: Cytokine release syndrome
PD-1: Programmed cell death 1
FACS: Fluorescence-activated cell sorting
PBS: Phosphate buffered saline
NS: Normal saline
FITC: Fluorescein isothiocyanate
PE: Phycoerythrin
APC: Allophycocyanin
BLI: Bioluminescence imaging
ELISA: Enzyme linked immunosorbent assay
ATCC: American Type Culture Collection
Raji-Luc: Raji cells stably expressing firefly luciferase
scFv: Single chain variable fragment
PBMC: Peripheral blood mononuclear cells
CFSE: Carboxyfluorescein diacetate succinimidyl ester
OMIM: Online Mendelian Inheritance in Man
PPI: Protein-protein interaction
DC: Degree Centrality
BC: Betweenness Centrality
CC: Closeness Centrality
KEGG: Kyoto Encyclopedia of Genes and Genomes
GO: Gene Ontology
DAVID: Database for Annotation, Visualization, and Integrated Discovery
RIN: RNA Integrity Number
DEGs: Differentially expressed genes
qRT-PCR: Real-time quantitative polymerase chain reaction
DS 2016: Discovery Studio 2016
NHL: Non-Hodgkin’s lymphoma
TP: T cell proliferation
PDIs: Plant-derived immunomodulators
OKT3: Soluble anti-human CD3 antibody
Ka: The association rate constant
Kd: The dissociation rate constant
KD: Affinity constants
NHS: Sulpho-*N*-hydroxysuccinimide
EDC: 1-ethyl-3-(3-dimethylaminopropyl) carbodiimide
